# The relationship between self-reported and registry-based data on use of psychoactive medications in postmenopausal women

**DOI:** 10.1186/1471-244X-13-180

**Published:** 2013-07-02

**Authors:** Päivi H Rauma, Heli Koivumaa-Honkanen, Heikki Kröger, Marjo T Tuppurainen, Jussi Kauhanen, Risto J Honkanen

**Affiliations:** 1 Social Pharmacy, School of Pharmacy, Faculty of Health Sciences, University of Eastern Finland (UEF), P.O. Box 1627, 70211, Kuopio, Finland; 2 Bone and Cartilage Research Unit, Surgery, Institute of Clinical Medicine, UEF, Kuopio, Finland; 3 Institute of Clinical Medicine, Psychiatry, UEF, Kuopio, Finland; 4 Institute of Clinical Medicine, Psychiatry, University of Oulu, Oulu, Finland; 5 Departments of Psychiatry; Kuopio University Hospital (KUH), Kuopio; South-Savonia Hospital District, Mikkeli; North Karelia Central Hospital, Joensuu; SOSTERI, Savonlinna; SOTE, Iisalmi; Lapland Hospital District, Rovaniemi, Finland; 6 Department of Orthopaedics, Traumatology and Hand Surgery, KUH, Kuopio, Finland; 7 Department of Obstetrics and Gynaecology, KUH, Kuopio, Finland; 8 Institute of Public Health and Clinical Nutrition, UEF, Kuopio, Finland

**Keywords:** Antidepressants, Menopause, Psychoactive drugs, Registers, Self-report, Validation studies

## Abstract

**Background:**

Self-report is commonly used as a source of information on the use of medicine. The aim of this study was to investigate the relationship between self-reported and register-based information on the use of psychoactive medication, especially in respect to antidepressants, and reasons of non-reporting.

**Methods:**

Study subjects (n = 11,031) originated from a population-based cohort of postmenopausal women born in 1932–41 from Eastern Finland who responded to a postal enquiry in 1999. Self-reported currently used prescribed medications were compared to the National prescription register data. Diuretics served as a reference for psychoactive medications.

**Results:**

Only 44% out of 1,638 women reported their use of psychoactive medication when compared to the prescription register within a 4-month time window preceding their response to enquiry. Altogether, 55% out of 777 women reported their use of antidepressants and 29% out of 861 reported their use of other psychoactive medications. In comparison 83% reported their use of diuretics. After excluding the occasional use, an increase in sensitivity by approximately 10 percentage points was seen regardless of the group of psychoactive medication. High use and history of work disability pension due to psychiatric cause were associated with a much higher likelihood of reporting psychoactive medication use (for antidepressants 70% and 81%, respectively).

**Conclusions:**

For research purposes, self-reported current use of psychoactive medication seems to be a sufficient indicator for regular use of antidepressants or in respect of use of any psychoactive medication, for subjects with severe psychiatric disease.

## Background

Self-report is commonly used as a source for information regarding medication exposure in epidemiological studies. This type of data is dependent on memory and maybe vulnerable to recall bias, but also unwillingness to report may play a role. Thus, for valid results, accurate information or at least knowledge of the degree of misreports is needed. The validity of self-reported medication use can be assessed by comparing self-reports to other data sources such as pharmacy data or medical records. Previous studies have investigated several different groups of medications [1-10]. For example, Klungel *et al.*[10] reported that approximately 70% of all medications currently used by patients with hypertension were correctly recalled in a self-administered questionnaire. However, the validity of self-reports has been found to vary extensively according to the type of medication taken [9,11-13]. The use of psychoactive medication has been shown to be less accurately reported than other medications [9,11,13].

Depression is one of the leading causes of disease burden in the world and a major public health problem also among the elderly [14]. The occurrence of depression is higher in women than men and its incidence peaks again after menopause [15]. There is limited validation data focusing solely [16-19] or among others [9,11-13,20-22] on the use of antidepressants or other psychoactive agents. Especially large cohort studies are rare [13,22]. Only few studies have investigated factors affecting accuracy of self-reported use of these medications [16,22]. Thus, more knowledge is needed regarding the extent and reasons associated with non-reporting of psychoactive medications, which could serve as a guide for future research.

The aim of the present study is to investigate among postmenopausal women from Eastern Finland, the agreement between self-reported use and registry-based data on purchased psychoactive medications, with particular reference to antidepressants. The causes for non-reports will also be investigated.

## Methods

### Study design and subjects

The study population (n = 11,031) for this cross-sectional population-based study was formed as follows: in 1989, a baseline postal enquiry was sent to all the 14,220 female residents born in 1932–41 of Kuopio Province, Finland (OSTPRE Study) [23,24]. The 10-year follow-up enquiry in 1999 was sent to 12,562 women alive and with known address out of the 13,100 baseline respondents. This enquiry was returned by 11,537 women. Out of these 11,537 women, 11,031 women responded to the question determining current medication usage in 1999. The study has been approved by the ethics committee of Kuopio University Hospital. Informed consent of the study subjects was obtained by postal enquiry.

### Questionnaire data

The study questionnaire included questions on marital status, residency (rural/city), height, weight, number of chronic health disorders, number of prescribed medications, use of hormone therapy (HT) (no/yes), life satisfaction (LS), physical activity (hours per week), smoking (no/yes) and consumption of beer, wine and distilled alcoholic beverages. The total use of alcohol was computed as grams (12 g/drink) per month and categorized (none/<360 g/≥360 g). Body mass index (BMI) was calculated as weight (kg)/height (m) squared and categorized (<25/25-29/≥30 kg/m^2^). LS was measured with a 4-item scale (range 4–20) [25-29]. It was used as a 3-category variable: 4–6 (satisfied), 7–11 (intermediate) and 12–20 (dissatisfied) based on the standard deviation of the mean [27]. Education (4-category) was asked on a subsample (n = 3,222) who underwent bone densitometry. All these factors were tested as covariates in multivariate analyses.

Participants were asked current medication usage: “Are you at the moment under medication prescribed by a doctor? Specify the names of the medications.” The reported medications were coded according to the Anatomical Therapeutic Chemical (ATC) code [30]. The ATC-categories investigated in the present study were: any psychoactive medications (N05, N06), antidepressants (N06A, N06CA) (with or without using other psychoactive medications) and other psychoactive medications (N05, N06B, N06CB, N06D) (without using antidepressants). In addition, “sole use of antidepressants” was also investigated. Purchasing of anti-dementia drugs (ATC: N06D) or psychostimulants/nootropics (N06B) during 2000–2005 according to National prescription register was used as memory variable (no/yes). Diuretics (C03), which are an independent, usual and regularly used medication group, were chosen to serve as a reference for psychoactive medications. Diuretics were used as follows: 1) any use (in tables), 2) together with psychoactive medication and 3) without psychoactive medication.

### Register data

The study questionnaire data was linked with the National prescription register and the National Register for Work Disability Pensions by personal identification codes. The prescription register from the Social Insurance Institution of Finland (KELA) contains data regarding all reimbursed prescription medicines purchased in any pharmacy in Finland. It does not include information on 1) medication use in hospitals or community nursing homes, where medicines are included in the services; 2) medications which are not reimbursable such as small packages costing under a fixed deductible share (i.e. 8.41 € in 1999) unless being a specially refunded medicine due to the selected chronic diseases including psychosis [31]. The defined daily doses (DDD) for medical products have been obtained from the Finnish Medicines Agency (FIMEA).

History of work disability pension up to the year 1996 (no/due to somatic cause/due to psychiatric cause) and medications purchased from a pharmacy within four or within 12 months before response to the enquiry in 1999 as DDD tertiles were used in the analysis. In Finland, only 3 months’ refunded medication can be purchased from pharmacy at a time. Thus, a 4-month time window preceding the date of response to enquiry was chosen for prescription register data to ensure that a person would purchase a medication at least once (if used continuously) [19]. In order to explore the agreement of self-report, also a 12-month time window preceding the enquiry and a 4-month time window after the enquiry were applied. For loss analysis, the index date for women who did not return the questionnaire was the date the questionnaire was sent.

### Statistical analyses

Statistical analyses were performed using the SPSS statistical package 17.0 for Windows (SPSS Inc., Chicago, IL, USA). Differences between study groups were examined with the Chi-squared test and the t-test for independent groups, or, in the case of variables not following a normal distribution, the non-parametric Mann–Whitney U-test. Agreement between the self-reported use and the National prescription register data as the golden standard were analyzed with sensitivity (i.e. recall rate or accuracy), specificity and Kappa-values (overall agreement).

To study factors affecting agreement of self-report and register data, logistic regression models were used. Only those women who according to the prescription register had purchased antidepressants or psychoactive medications were selected. Self-report (no/yes) was the outcome measure (i.e. dependent variable). Multivariate analysis was used to assess the independent effect of certain personal and health-related factors. Covariates were chosen into models if they were significantly associated with outcome. In the multivariate model 1, age, marital status, BMI, alcohol consumption and LS were covariates. In model 2, additional covariates included history of work disability pension up to the year 1996 and number of self-reported prescribed medications as well as amount of purchased psychoactive medications from the prescription register.

## Results

### Characteristics

According to the prescription register, 1,638 (14.8%) of the 11,031 participants, had purchased psychoactive medications during the four months preceding the self-report. A total of 777 (7.0%) women had purchased antidepressants with or without other psychoactive medications, while 861 (7.8%) had purchased other psychoactive medications than antidepressants. Out of the 777 antidepressant users, 393 (3.6%) were “sole antidepressant users”, while 384 women also used other psychoactive medications. Among these 384 women, antipsychotics were used by 123 women, anxiolytics by 198 women and hypnotics or sedatives by 184 women. In the entire group of users of other psychoactive medications (n = 861), antipsychotics were used by 147 women, anxiolytics by 315 and hypnotics or sedatives by 513 women. Users of psychoactive medications based on prescription register differed from non-users for all the study variables (Table 1). Users had more diseases and were less satisfied with their lives, while non-users were better off in terms of smoking and BMI, but not in terms of alcohol use. Of the entire study population, 22.7% had a history of work disability pension, this proportion being highest among users of antidepressants (43.2%).

**Table 1:** Characteristics of study population (n = 11,031) by use of medication based on the National prescription register

	**All (n = 11,031)**		**No psychoactive medication (n = 9,393)**		**Any psychoactive medication users (n = 1,638)**		**Antidepressants users (n = 777)**		**Other psychoactive medication users without antidepressants (n = 861)**		**Diuretics users **^**b **^**(n = 1,249)**	
**Characteristic**												
Continuous	mean	(SD)	mean	(SD)	mean	(SD)	mean	(SD)	mean	(SD)	mean	(SD)
Age (years)	62.3	(2.9)	62.2	(2.9)	62.5	(3.0)***	62.3	(3.0)	62.7	(2.9)***	62.9	(2.9)***
BMI (kg/m^2^)	27.6	(4.7)	27.5	(4.6)	28.3	(5.2)***	28.4	(5.2)***	28.2	(5.2)***	30.5	(5.5)***
Number of prescribed medications	2.1	(2.0)	1.8	(1.9)	3.3	(2.2)***	3.5	(2.2)***	3.1	(2.2)***	4.0	(2.2)***
Number of diseases	2.4	(1.8)	2.3	(1.8)	3.2	(2.0)***	3.2	(2.0)***	3.2	(1.9)***	3.5	(2.0)***
Self-rated LS ^a^	8.1	(2.8)	7.9	(2.6)	9.4	(3.5)***	9.9	(3.6)***	9.0	(3.3)***	8.5	(3.1)***
Categorical	n	(%)	n	(%)	n	(%)	n	(%)	n	(%)	n	(%)
Disability pension ^c^	2,500	(22.7)	1,854	(19.7)	646	(39.5) ***	328	(43.2) ***	318	(36.9)***	416	(33.3)***
HT use	2,405	(21.8)	2,010	(21.4)	395	(24.1)*	215	(27.7)***	180	(20.9)	224	(17.9)***
Smoking	908	(8.2)	695	(7.4)	213	(13.0)***	90	(11.6) ***	123	(14.3) ***	87	(7.0)
Use of alcohol	5,487	(51.4)	4,749	(52.1)	738	(47.0) ***	344	(46.3) **	394	(47.6)*	540	(45.6)***

Abbreviations: *BMI*, body mass index; *HT*, hormone therapy; *SD*, standard deviation.

*P value < 0.05, **P < 0.005, ***P ≤ 0.001 difference from non-users.

^a^ Self-rated life satisfaction, range 4 to 20, higher score indicating lower life satisfaction.

^b^ The group includes all diuretics users.

^c^ History of work disability pension up to 1996.

### Sensitivity between self-reported and prescription register data

Self-reported use of medication was compared to the prescription register data within a 4-month time window preceding the response to enquiry (Tables 2 and 3). The sensitivity of self-reports was 44.2% for all psychoactive medication. It was higher for use of antidepressants (54.8%) than for use of other psychoactive medications (29.0%). For sole antidepressants users, the proportion was 50.1%. Altogether, 66 women reported use of antidepressants and 152 women reported use of any psychoactive medication, though they had not purchased the medication within the 4-month time window preceding the self-report (Table 2). For the preceding 12 months prior to the self-report, these figures were 12 and 40 persons, respectively.

**Table 2:** Comparison of self-reports and the National prescription register data for psychoactive medication and diuretics

	**Self-reported**							
	**Any psychoactive medication**		**Antidepressants**		**Other psychoactive medication without antidepressants**		**Diuretics **^**b**^	
	*yes*	*no*	*yes*	*no*	*yes*	*no*	*yes*	*no*
Prescription register								
*Yes*^*a*^	724	914	426	351	250	611	1,039	210
*No*	152	9,241	66	10,188	344	9,826	197	9,585
Sensitivity	0.44		0.55		0.29		0.83	
Specificity	0.98		0.99		0.97		0.98	
κappa	0.527*		0.652*		0.299*		0.815*	

* P value < 0.001.

^a^ Purchased the given medication within four months before response to postal enquiry.

^b^ The group includes all diuretics users.

**Table 3:** Sensitivity (%) of self-reported psychoactive medication use by characteristics of study subjects compared to the National prescription register data during the four months preceding the self-report

**Use of psychoactive medication according to the National prescription data**									
	**Any**			**Antidepressant**			**Other**		
**Characteristics**	**%**	**(n)**	***P *value**^**a**^	**%**	**(n)**	***P *value**^**a**^	**%**	**(n)**	***P *value**^**a**^
Self-reported use of psychoactive medication	44.2	(724/1,638)		54.8	(426/777)		29.0	(250/861)	
Age (years)			< 0.001			0.254			< 0.001
57 - 62	49.3	(429/871)		56.6	(248/438)		36.0	(156/433)	
63 - 68	38.5	(295/767)		52.5	(178/339)		22.0	(94/428)	
Marital status			< 0.001			0.021			< 0.001
Single	64.3	(99/154)		70.0	(56/80)		47.3	(35/74)	
Married / cohabitating	42.3	(413/977)		54.8	(251/458)		26.2	(136/519)	
Divorced	50.0	(87/174)		51.7	(45/87)		40.2	(35/87)	
Widoved	36.7	(114/311)		48.9	(69/141)		22.9	(39/170)	
Body mass index (kg/m^2^)			0.011			0.195			0.115
< 25	41.1	(170/414)		50.8	(93/183)		27.7	(64/231)	
25 – 29.99	42.1	(262/623)		54.4	(162/298)		27.1	(88/325)	
≥ 30	49.8	(240/482)		59.6	(137/230)		34.5	(87/252)	
Alcohol consumption (g/month)			< 0.001			0.075			< 0.001
None	48.1	(400/832)		57.1	(228/399)		33.9	(147/433)	
< 360	38.0	(264/695)		51.3	(164/320)		21.9	(82/375)	
≥ 360	51.2	(22/43)		37.5	(9/24)		63.2	(12/19)	
Number of self-reported diseases			0.911			0.365			0.670
0	45.9	(34/74)		44.4	(16/36)		34.2	(13/38)	
1 − 2	43.4	(258/594)		53.5	(154/288)		29.1	(89/306)	
≥ 3	44.0	(410/931)		56.1	(245/437)		27.7	(137/494)	
Smoking			0.190			0.410			0.075
no	43.6	(621/1,425)		54.3	(373/687)		27.9	(206/738)	
yes	48.4	(103/213)		58.9	(53/90)		35.8	(44/123)	
HT use			0.727			0.872			0.357
no	44.0	(546/1,240)		55.2	(309/560)		29.9	(203/680)	
yes	45.1	(178/395)		54.4	(118/217)		26.1	(47/180)	
Life satisfaction			0.016			0.222			0.472
satisfied	42.5	(102/240)		63.0	(58/92)		25.6	(53/207)	
intermediate	42.0	(402/958)		53.1	(229/431)		28.7	(142/494)	
dissatisfied	50.1	(212/423)		54.9	(134/244)		31.4	(45/143)	
Number of self-reported prescribed medication			<0.001			<0.001			<0.001
0	0.0	(0/110)		0.0	(0/35)		0.0	(0/75)	
1 − 2	34.2	(202/590)		40.8	(115/282)		24.4	(75/308)	
≥ 3	55.7	(522/938)		67.6	(311/460)		36.6	(175/478)	
Amount of purchased medication ^b^			< 0.001			< 0.001			< 0.001
low	23.4	(120/513)		35.4	(85/240)		12.9	(40/309)	
moderate	42.5	(259/610)		56.8	(151/266)		39.6	(82/207)	
high	67.0	(345/515)		70.1	(190/271)		37.1	(128/345)	
History of work disability pension			< 0.001			< 0.001			< 0.001
no	38.7	(384/992)		51.4	(231/449)		24.3	(132/543)	
somatic cause	39.1	(181/463)		47.4	(99/209)		25.6	(65/254)	
psychiatric cause	86.9	(159/183)		80.7	(96/119)		82.8	(53/64)	

Abbreviations: *HT*, hormone therapy.

^a^*P* value from the chi-square test between the subgroups.

^b^ Tertile cutoffs for amount of use (DDD/4 months): psychoactive medication <34 (low), 34–100 (moderate), >100 (high), antidepressants <33, 33–99.9, ≥100, other psychoactive medication <33, 33–99.99, ≥100.

Self-reported use of diuretics covered a much higher percentage (83.2%) of the register-based data than use of psychoactive medication. Users of psychoactive medication also reported their use of diuretics less accurately (76.0%) than non-users (85.1%) (*P* = 0.001).

The sensitivity in use of psychoactive medication between the two data sources was better when only regular use was considered by taking into account medication purchased within four months both before and after the enquiry: the proportion of those reporting their use of medication was, thus, 55% for all psychoactive medications and 65% for antidepressants.

The exclusion of the low use tertile (i.e. occasional/irregular use) and the widening of the register data time window improved the sensitivity between self-report and register data. Thus, the sensitivity of self-reported regular use of antidepressants was 71.3%, if the medication purchase precondition was four months before and after the response to enquiry. The proportion of false positive self-reports decreased from 13.4 to 2.5%, if the time window was widened from 4 to 12 months before the response to enquiry.

Sensitivity and specificity of self-reports were 0.44 and 0.98 respectively, for psychoactive medication and 0.83 and 0.98 respectively for diuretics (Table 2). Kappa-values were 0.53 for all psychoactive medication, 0.65 for antidepressants, 0.30 for other psychoactive medications and 0.82 for diuretics (all *P* < 0.001). Sensitivity of self-reported psychoactive medication increased markedly according to the amount of use (Table 3).

### Factors affecting sensitivity of self-report

The amount of purchased psychoactive medications (DDDs/four months), history of work disability pension due to psychiatric cause and the number of any self-reported prescribed medications as well as being unmarried were strongly associated with higher sensitivity between self-reported and register-based data regardless of type of medication (Table 3). Among users of any psychoactive medication also life dissatisfaction and higher BMI were associated with better sensitivity.

In multivariate models, greater alcohol consumption was associated with lower sensitivity for use of antidepressants (odds ratios, OR_model1_ = 0.39, *P* = 0.045 and OR_model2_ = 0.34, *P* = 0.043) (Table 4). In respect to marital status, those who are single had higher sensitivity than others (Tables 4 and 5). Higher number of any self-reported prescribed medication, higher use of antidepressants or any psychoactive medication according to prescription register and work disability pension due to psychiatric cause (model 2) were all associated independently with higher sensitivity, while work disability pension due to somatic cause was associated with lower sensitivity.

**Table 4:** Factors related to the sensitivity of self-reported use of antidepressants compared to register-based data (n = 777) by logistic models

	**Univariate model**			**Multivariate model 1 **^**c **^**(n = 679)**			**Multivariate model 2 **^**c **^**(n = 679)**		
**Variables**	**OR**	**95% CI**	***P *value **^**a**^	**OR**	**95% CI**	***P *value **^**a**^	**OR**	**95% CI**	***P *value **^**a**^
Age (years)	0.97	0.93, 1.02	0.241	0.97	0.93, 1.03	0.329	0.97	0.92, 1.03	0.312
Marital status									
single	1.0			1.0			1.0		
cohabitation / married	0.52	0.31, 0.87	0.012	0.47	0.27, 0.81	0.007	0.58	0.32, 1.06	0.076
divorced	0.46	0.24, 0.87	0.017	0.46	0.23, 0.92	0.029	0.52	0.24, 1.12	0.097
widoved	0.41	0.23, 0.73	0.003	0.40	0.21, 0.76	0.005	0.53	0.26, 1.06	0.073
Body mass index (kg/m^2^)									
< 25	1.0			1.0			1.0		
25-29	1.15	0.80, 1.67	0.450	1.31	0.89, 1.93	0.168	1.14	0.74, 1.75	0.562
≥ 30	1.43	0.96, 2.11	0.076	1.50	0.99, 2.25	0.054	0.87	0.55, 1.38	0.551
Alcohol consumption (g/month)									
none	1.0			1.0			1.0		
< 360	0.79	0.59, 1.06	0.115	0.84	0.61, 1.16	0.287	0.91	0.64, 1.29	0.593
≥ 360	0.45	0.19, 1.05	0.066	0.39	0.16, 0.98	0.045	0.34	0.12, 0.97	0.043
Life satisfaction									
satisfied	1.0			1.0			1.0		
intermediate	0.67	0.42, 1.06	0.084	0.61	0.37, 1.01	0.054	0.70	0.41, 1.22	0.212
dissatisfied	0.71	0.44, 1.17	0.180	0.72	0.42, 1.24	0.235	0.74	0.41, 1.34	0.313
Number of prescribed medications (continuous)	1.38	1.28, 1.49	< 0.001	–			1.37	1.25, 1.50	< 0.001
Amount of purchased antidepressants ^b^									
low	1.0			–			1.0		
moderate	2.39	1.67, 3.43	< 0.001	–			2.56	1.68, 3.89	< 0.001
high	4.28	2.95, 6.20	< 0.001	–			3.81	2.51, 5.79	< 0.001
History of work disability pension									
no	1.0			–			1.0		
somatic cause	0.85	0.61-1.18	0.330	–			0.63	0.42, 0.95	0.026
psychiatric cause	3.94	2.41-6.44	< 0.001	–			2.55	1.46, 4.46	0.001

Abbreviations: *CI*, confidence interval; *OR*, odds ratio.

^a^*P* value from the chi-square test.

^b^ Tertile cutoffs for amount of use (DDD/4 months): <33 (low), 33–99.9 (moderate), ≥100 (high).

^c^ Multivariate models include all the variables in the column.

**Table 5:** Factors related to the sensitivity of self-reported use of any psychoactive medication compared to register-based data (n = 1,638) by logistic models

	**Univariate model**			**Multivariate model 1 **^**c **^**(n = 1,448)**			**Multivariate model 2 **^**c **^**(n = 1,446)**		
**Variables**	**OR**	**95% CI**	***P *value **^**a**^	**OR**	**95% CI**	***P *value **^**a**^	**OR**	**95% CI**	***P *value **^**a**^
Age (years)	0.93	0.90, 0.96	< 0.001	0.93	0.90, 0.97	< 0.001	0.94	0.91, 0.98	0.006
Marital status									
single	1.0			1.0			1.0		
cohabitation/married	0.41	0.29, 0.58	< 0.001	0.42	0.29, 0.62	< 0.001	0.48	0.31, 0.73	0.001
divorced	0.56	0.36, 0.87	0.009	0.55	0.34, 0.89	0.015	0.60	0.35, 1.03	0.064
widoved	0.32	0.22, 0.48	< 0.001	0.34	0.22, 0.53	< 0.001	0.38	0.23, 0.62	< 0.001
Body mass index (kg/m^2^)									
< 25	1.0			1.0			1.0		
25-29	1.04	0.81, 1.34	0.751	1.15	0.88, 1.49	0.317	1.00	0.74, 1.34	0.989
≥ 30	1.42	1.09, 1.86	0.009	1.46	1.11, 1.94	0.008	0.95	0.69, 1.31	0.770
Alcohol consumption (g/month)									
none	1.0			1.0			1.0		
< 360	0.74	0.58, 0.95	0.018	0.68	0.55, 0.85	0.001	0.80	0.63, 1.03	0.079
≥ 360	0.63	0.49, 0.81	< 0.000	0.87	0.45, 1.67	0.672	0.89	0.42, 1.87	0.749
Life satisfaction									
satisfied	1.0			1.0			1.0		
intermediate	0.98	0.74, 1.30	0.880	0.99	0.73, 1.35	0.954	1.04	0.73, 1.47	0.837
dissatisfied	1.36	1.00, 1.87	0.059	1.32	0.93, 1.87	0.118	1.20	0.81, 1.78	0.371
Number of prescribed medications (continuous)	1.34	1.27, 1.40	< 0.001	–			1.32	1.25, 1.40	< 0.001
Amount of purchased psychoactive medication^b^									
low	1.0			–			1.0		
moderate	2.42	1.86, 3.13	< 0.001	–			1.85	1.38, 2.47	< 0.001
high	6.65	5.05, 8.75	< 0.001	–			4.12	3.04, 5.60	< 0.001
History of work disability pension									
no	1.0			–			1.0		
somatic cause	1.02	0.81, 1.27	0.889	–			0.66	0.50, 0.86	0.003
psychiatric cause	10.49	6.70, 16.42	< 0.001	–			6.53	3.90, 10.92	< 0.001

Abbreviations: *CI*, confidence interval; *OR*, odds ratio.

^a^*P* value from the chi-square test.

^b^ Tertile cutoffs for amount of use (DDD/4 months): <34 (low), 34–100 (moderate), >100 (high).

^c^ Multivariate models include all the variables in the column.

Among women using any psychoactive medication (n = 1,638) (Table 5), improved sensitivity between self-report and register-based data was associated with younger age (all models), but not with residence or physical activity nor was it related to education in the sample of 3,222 women, or to registry-based data on subsequent use of medication for dementia. However, only 92 women out of the entire study population had purchased anti-dementia medication in 2000–2005. Obesity was associated with better sensitivity in the use of any psychoactive medication (model 1), but the inclusion of the number of self-reported prescribed medications and history of work disability variables into the model totally abolished this association (Table 5).

Also among women with register-based sole antidepressants use (n = 393), a greater number of any prescribed medication (OR = 1.33, *P* < 0.001), amount of purchased antidepressants (OR_moderate use_ = 2.89, *P* < 0.001 and OR_high use_ = 3.57, *P* < 0.001) and history of work disability due to psychiatric cause (OR = 4.04, *P* = 0.002) remained correlates of self-report in the multivariate model 2 (Cf. Table 4).

### Loss analysis

According to the prescription register, the 1,025 women who did not return the questionnaire, purchased (during the four months preceding the follow-up enquiry in 1999) more often than the responders who reported any psychoactive medication (23.3 vs. 14.8%, *P* < 0.001), antidepressants (9.3 vs. 7.0%, *P* = 0.010) or other psychoactive medications (14.0 vs. 7.8%, *P* < 0.001). The corresponding proportions, for the 506 women who returned the questionnaire but did not answer the medication question were similar with those of the responders (15.2, 6.3 and 8.9%, respectively). Correction of non-response to the enquiry or the medication question increased the population estimate for use of any psychoactive medication from 14.8 to 15.5%, use of antidepressants from 7.0 to 7.2% and use of other psychoactive medication from 7.8 to 8.4%.

## Discussion

Postmenopausal women are at increased risk of depression. This study investigated agreement between self-reported use of psychoactive medication and the National prescription register data and possible related factors among postmenopausal women. The sensitivity of self-report was 44% for any psychoactive medication and 55% for antidepressants, while it was over 80% for diuretics. However, higher use and history of work disability pension due to psychiatric cause increased sensitivity strongly.

Methodological differences such as selected golden standard, interview vs. enquiry method or structure and wording of questions might be reasons for the differences in the results between studies. Other Finnish studies have previously found high overall agreement (ad 0.87) for antidepressants [18,19] and antipsychotics [18,19,22] with the same golden standard as in this study. This agreement difference might be due to longer purchase time window [18,22], younger population [18,22] or the interview vs. enquiry method and prescriptions brought to the interview [18,19]. A large study by Nielsen *et al.*[13] with home interview but pharmacy records as a reference, found self-report sensitivity of antidepressant use (56%) similar to ours. Also Boudreau *et al*. [9] found lower sensitivity for antidepressants (44-66%) when using interview with specific medication questions. However, higher sensitivities for self-report of antidepressants and psychoactive medication (70-89%) have generally been obtained with different methodologies; such as medical records [12] or physician-reported data as golden standard [16], when asked “never/ever” use [16] or a direct question on medication use [17,21] or when prescriptions were brought to the interview [18]. On the other hand, Nielsen *et al.*[13] also found low sensitivity for psycholeptics (38%) and especially for hypnotics and sedatives (27%). Thus, the low sensitivity in our study (29%) for the group of other psychoactive medication might be due to the high proportion of users of hypnotics and sedatives in our study sample. Similar to our study, moderate overall agreement (>0.50) for psycholeptics [13] (vs. psychoactive medication) and even better for antidepressants reporting [12,13,16,17,22] has been shown by others.

In the present study, users of psychoactive medication showed lower sensitivity for diuretics than non-users, but they reported the use of diuretics better than the use of psychoactive medication. Also in previous studies, the use of psychoactive medication has been less accurately reported than other medications [9,11,13]. Interestingly, however, a lower sensitivity in respect to psychoactive medication in this study was linearly related to less severe psychiatric problems (i.e. no work disability pension due to psychiatric cause) and lower use of psychoactive medication. One explanation may simply be that psychoactive medicines such as hypnotics are not used as regularly as diuretics. One might just not feel that occasional use is important enough to be mentioned in a questionnaire or the medicine may not be used at the moment or at all after purchase. Lin *et al.*[32] found that 28% of patients discontinued their antidepressant medication during the first month of therapy and 44% by the third month of therapy. Longer and regular use may increase recall or reduce self-perceived shame or unwillingness to report the use [10,11,16,33].

In Finland the use of antidepressants doubled between 1999–2009 [34], which might decrease the unwillingness to report use of medication. However, our study subjects increased less their use of antidepressants between 1999–2009 and the sensitivity of self-report of antidepressants in 2009 was similar to that in 1999 (data not shown). It seems that regular use of antidepressants and/or long-term severe mental illness (as indicated by work disability pension) are the most important correlates of sufficient self-report.

In general, several factors may affect the agreement between self-reports and register-based data. Obesity has been previously positively associated with agreement when self-reports have been compared to medical records [12], but higher BMI is associated also with depression [35]. Thus, after including the amount of antidepressants or psychoactive medication (model 2), the significance of BMI in respect to sensitivity disappeared. Lower income [5,16] and education level [3,5,10,22] have been associated with poorer agreement, but Sjahid *et al.*[6] found no differences according to educational or socio-economic status. Homogeneity of our cohort may have played a role in some of the factors. In our female sample, only a small number of women had a higher education, which may have contributed to the non-significance of education. Older age has [11,36] or has not [6] been associated with decreased validity of self-reports, while narrow age window in our study (57–68 years) might explain the found minimal age effect. In this study, being single was associated with the highest sensitivity in reporting psychoactive medication use, while in another Finnish cohort born in 1966 [22] the same was true for the married subjects. Lastly, even if Van den Brandt *et al.*[11] study suggested a decrease in recall along with increasing number of prescribed medications, in our study higher number of any prescribed medication currently in use was associated with better sensitivity. Stability of disease status indicated by history of work disability pension and amount of psychoactive medication in our study seemed to be more important positive determinants of agreement than putative (negative) effects of mental disease or its medication.

The strengths of this study include a large population-based cohort, high response rate and utilization of the National prescription register, which has a coverage of 97% and ability to detect also slight (i.e. irregular) use [31]. The narrow age range increased the homogeneity of the sample, but provides data on important period around retirement. Lastly, our results apply to postmenopausal women, a risk group for depression, and a base for future growing elderly population. Our method of asking current use of any prescribed medications may give lower agreement when compared to the results obtained by never-ever -type questions. Since the prescription register as a reference indicates only the medication purchased and not the actual use, it possesses a possibility to non**-**agreement, but this inaccuracy was minimized by checking also purchase of medication after enquiry.

## Conclusions

Less than half of the women reported their use of any psychoactive medication and just over half their use of antidepressants compared to register-based data on purchased medication. The sensitivity of self-report increased considerably with higher use, with a history of work disability pension due to psychiatric cause or when occasional use was excluded. In conclusion, for research purposes, self-reported current medication use seems in general to be a sufficient indicator of regular use of antidepressants or in respect of use of any psychoactive medication, for those with severe psychiatric disease. However, prescription register data is more reliable for those using these medications irregularly and with less severe mental disorders.

### Ethical standards

This study was approved by the ethics committee of Kuopio University Hospital, Finland.

## Abbreviations

ATC: Anatomical-Therapeutic-Chemical; BMI: Body mass index; CI: Confidence interval; DDD: Defined daily doses; HT: Hormone therapy; LS: Life satisfaction; OR: Odds ratio; OSTPRE: Kuopio Osteoporosis Risk Factor and Prevention Study; SD: Standard deviation.

## Competing interests

All authors declare that they have no conflicts of interest.

## Authors’ contributions

PR, HK-H and RH designed the study. PR conducted data analysis, data interpretation, and the writing of the manuscript. HK-H, RH and JK were responsible for the data and contributed to data interpretation. RH, MT and HK were responsible for the original OSTPRE study design. All the authors contributed to the revision of the manuscript and approved the final version.

## Pre-publication history

The pre-publication history for this paper can be accessed here:

http://www.biomedcentral.com/1471-244X/13/180/prepub
